# Utility of white matter disease and atrophy on routinely acquired brain imaging for prediction of long-term delirium risk: population-based cohort study

**DOI:** 10.1093/ageing/afab200

**Published:** 2021-11-13

**Authors:** Sarah T Pendlebury, Ross J Thomson, Sarah J V Welch, Wilhelm Kuker, Peter M Rothwell, for the Oxford Vascular Study

**Affiliations:** Wolfson Centre for Prevention of Stroke and Dementia, Wolfson Building, Nuffield Department of Clinical Neurosciences, John Radcliffe Hospital and the University of Oxford, Oxford, UK; NIHR Oxford Biomedical Research Centre, Oxford University Hospitals NHS Foundation Trust, Oxford, UK; Departments of General (Internal) Medicine and Geratology, Oxford University Hospitals NHS Foundation Trust, Oxford, UK; Department of Cardiology, Royal Free Hospital, London, UK; Wolfson Centre for Prevention of Stroke and Dementia, Wolfson Building, Nuffield Department of Clinical Neurosciences, John Radcliffe Hospital and the University of Oxford, Oxford, UK; Wolfson Centre for Prevention of Stroke and Dementia, Wolfson Building, Nuffield Department of Clinical Neurosciences, John Radcliffe Hospital and the University of Oxford, Oxford, UK; Wolfson Centre for Prevention of Stroke and Dementia, Wolfson Building, Nuffield Department of Clinical Neurosciences, John Radcliffe Hospital and the University of Oxford, Oxford, UK

**Keywords:** delirium, stroke, transient ischemic attack (TIA), brain imaging, older people

## Abstract

**Background:**

brain imaging done as part of standard care may have clinical utility beyond its immediate indication. Using delirium as an exemplar, we determined the predictive value of baseline brain imaging variables [white matter changes (WMC) and atrophy] for delirium risk on long-term follow-up after transient ischemic attack (TIA)/stroke in a population-based cohort study.

**Methods:**

surviving TIA/stroke participants in the Oxford Vascular Study (OXVASC) were assessed prospectively for delirium during all hospitalisations over 6 months (2013–14). Using logistic regression, independent associations were determined between baseline OXVASC computed tomography or magnetic resonance brain imaging measures of WMC and cerebral atrophy (none/mild versus moderate/severe) and delirium adjusted for age, sex, baseline stroke severity, depression, illness severity and pre-admission cognition.

**Results:**

among 1,565 TIA/stroke survivors with 194 hospital admissions (158 patients, mean/standard deviation age at admission = 79.2/11.5 years), delirium occurred in 59 (37%). WMC and atrophy on baseline imaging were associated with delirium [odds ratio (OR) = 3.41, 1.21–5.85, *P* = 0.001 and OR = 2.50, 1.23–5.08, *P* = 0.01 (unadjusted) and OR = 2.67, 1.21–5.85, *P* = 0.02 and OR = 2.18, 1.00–4.73, *P* = 0.05 (adjusted age and sex)]. Associations were strengthened when analyses were restricted to patients hospitalised within 5 years of baseline brain imaging [OR = 6.04, 2.39–15.24, *P* < 0.0001 and OR = 4.64, 1.46–14.82, *P* = 0.009 (unadjusted)] but only WMC remained significant after adjustment for all covariates including pre-admission cognition (OR = 4.83, 1.29–18.13, *P* = 0.02 for Mini-Mental State Examination and OR = 5.15, 1.26–21.09, *P* = 0.02 for Montreal Cognitive Assessment).

**Conclusions:**

WMC and atrophy on brain imaging done up to 5 years earlier predicted delirium and may have clinical utility in risk stratification. Associations with WMC but not atrophy were independent of pre-admission cognitive impairment.

## Key Points

- Brain imaging done as part of standard care may have clinical utility beyond its immediate indication.
- White matter changes and cerebral atrophy on brain imaging predict delirium occurring on long-term follow-up.
- Associations between white matter changes and delirium were independent of pre-admission cognition.
- Delirium risk prediction may be strengthened by inclusion of routinely acquired brain imaging markers.

## Introduction

Brain imaging is frequently performed for both clinical and research purposes and rates continue to rise with population ageing [1]. Routinely acquired brain scans represent an underused resource: for example, atrophy and cerebrovascular disease are associated with cognitive and functional decline and might be useful in clinical prediction models [2, 3]. Using delirium as an exemplar, we aimed to examine the predictive value of routinely acquired brain imaging for future delirium in the hospitalised population.

Delirium is prevalent in the acute hospital affecting over 40% of the oldest old in acute medicine wards and 50% of those with hip fracture and is associated with poor outcomes [4, 5]. Delirium risk is increased with prior cognitive impairment but cognitive problems may be unrecognised prior to admission [6, 7] impacting delirium recognition and prevention [8]. Delirium risk stratification to target prevention and intervention might be improved by incorporating measures of white matter changes (WMC) and atrophy, both of which are associated with reduced cerebral reserve and cognitive impairment [3, 9]. Use of existing previously acquired routine brain imaging data might prove cost-effective and would leverage previous investment in healthcare services. With the advent of electronic patient records, brain imaging data could eventually be incorporated into prediction algorithms to highlight at-risk patients in real time [8].

In a population-based cohort of transient ischemic attack (TIA)/stroke, we determined whether WMC burden and cerebral atrophy at baseline predicted delirium on long-term follow-up. We studied TIA/stroke patients since they undergo brain imaging at the time of the event and have high rates of small vessel disease and delirium during general hospital admission [4, 5, 10].

## Methods

### Oxford Vascular Study

Patients in the current study were participants with TIA or stroke previously recruited into the Oxford Vascular Study (OXVASC, 2002), an ongoing longitudinal population-based cohort study of all acute vascular events occurring within a defined population of 92,728 covered by nine primary care practices in Oxfordshire, UK [3, 11]. The study is approved by the local research ethics committee. Informed written consent (or assent from relatives) is obtained for baseline and follow-up interviews and indirect follow-up using medical records and death certificate data.

Patients with TIA/stroke resident within the OXVASC study population are ascertained through a combination of hot and cold pursuit [11]. TIA and stroke are defined clinically by the World Health Organization criteria. Baseline brain and vascular imaging is performed and all cases are reviewed by a senior vascular neurologist (P.M.R.). Patient data are collected by interview using a standardised form and from general practitioner records. Follow-up interviews are done at 1 and 6 months and 1, 5 and 10 years in the outpatient clinic or by home visit. Cognitive testing is done at baseline and all follow-ups using ≥one of Mini-Mental State Examination (MMSE [12]) and Montreal Cognitive Assessment (MoCA [13, 14]). Index stroke severity is measured using the National Institutes of Health Stroke Scale (NIHSS).

### Measures of WMC burden, cerebral atrophy

WMC severity was measured on either computed tomography (CT) or magnetic resonance imaging (MRI) brain scans done at the time of the index cerebrovascular event as described previously [10]. In the early years of the OXVASC study, CT was the first line baseline brain imaging modality. In later periods, MRI was predominantly used. WMC was graded according to severity (none, mild, moderate or severe) of the Blennow scale for CT scans, and a modified version of the Fazekas scale, for MRI scans. In a previous study on 416 OXVASC patients with both modalities of imaging, we demonstrated good agreement between CT versus MRI measures of severity of WMC (kappa = 0.72) and this was similar to the inter-rater agreement for severity of WMC within a given imaging modality [10]. Atrophy was categorised as none, mild, moderate or severe by an experienced neuroradiologist (W.K.) who performed all such assessments throughout the OXVASC study, rather than by visual rating scales that are complex and developed for MRI rather than CT [15, 16].

### Prospective ascertainment of delirium

**Figure 1:**
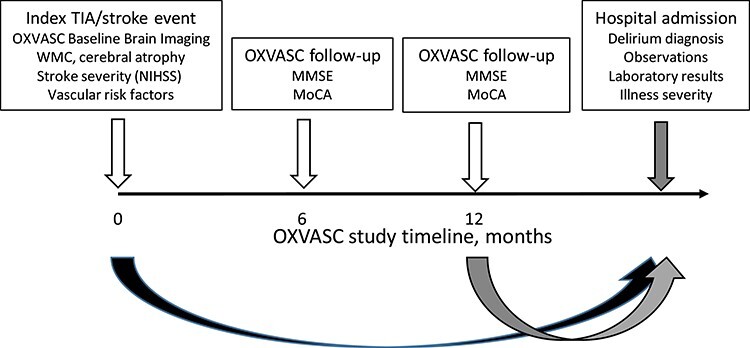
Schematic diagram showing the OXVASC study baseline TIA/stroke event and longitudinal follow-up (only the 6- and 12-month follow-ups are shown for illustration) with a subsequent hospital admission occurring after the 12-month follow-up as an example. WMC and cerebral atrophy measures are acquired from the baseline brain imaging done at the time of the index TIA/stroke according to standard OXVASC study methods. Time elapsed since baseline assessment to hospital admission is shown by the black curved arrow. MMSE and MoCA are acquired at each OXVASC follow-up assessment. Time elapsed since OXVASC follow-up assessment to hospital admission is shown by the grey curved arrow. At hospital admission, delirium diagnosis is ascertained together with measures of illness severity.

All TIA and stroke participants in OXVASC surviving on 1 October 2013 were included in the current study (Figure 1). Subsequent hospital admissions for any reason were prospectively identified using a method of hot pursuit (see below) from October 2013 to April 2014 at the Oxford University Hospitals NHS Foundation Trust (OUHFT) and the Abingdon Community Hospital Emergency Medicine Unit (EMU). Patients were assessed as soon as possible after admission by members of the OXVASC study team (S.J.V.W., R.J.T.).

The OUHFT provides acute services for the population of ~660,000 in Oxfordshire including for all patients in the OXVASC study primary care practices. OXVASC patients requiring acute hospital care for any reason are admitted either to OUHFT or to Abingdon EMU. Delirium was ascertained prospectively using the gold standard clinical diagnosis rather than from retrospective hospital administrative diagnostic (ICD-10) coded data since the latter are insensitive [17]. To identify OXVASC participants admitted to hospital over the study period, we conducted daily searches of electronic (OUHFT) and paper records (EMU) for all new admissions excluding day case procedures and cross-checked these against the register of OXVASC participants.

Delirium diagnosis was based on the DSM-IV [18] criteria (see Appendices). According to local protocols, all patients admitted to the OUHFT or EMU who are aged ≥70 or <70 years with confusion/altered behaviour or brain at-risk (e.g. stroke, Parkinson’s disease) have the OUHFT cognitive screen delivered via a standard admission proforma [4, 17]. The OUHFT cognitive screen includes the 10-point Abbreviated Mental Test score [19] together with the Confusion Assessment Method [20] for delirium and documentation of known dementia diagnosis. In patients discharged before assessment, diagnosis was made after review of all available medical records including the OUHFT cognitive screen. The diagnosis of delirium was made if the DSM-IV criteria were considered fulfilled by S.T.P., an experienced physician, after consideration of all available information including discussion with the OXVASC study team including interviews with staff members and review of the medical notes as described previously [4].

Admission physiological parameters (pulse, temperature and respiratory rate) and white cell count (WCC) were taken from the patient’s chart. Illness severity was defined by presence of the systemic inflammatory response syndrome (SIRS) in which two or more of heart rate > 90 beats/min, temperature < 36 or >38°C, respiratory rate > 20 breaths/min, WCC <4 × 109 or >12 × 109 cells per litre are present [21].

### Statistical analysis

Baseline characteristics of patients with any admission with delirium versus those without delirium were compared using *t*-test and analysis of variance, as appropriate, for continuous variables and chi square for categorical variables. MMSE and MoCA cognitive test scores were obtained from the most recent OXVASC study follow-up done prior to the hospital admission episode together with OXVASC study dementia diagnosis (Figure 1).

In order to maximise generalisability and applicability of brain imaging variables for delirium risk prediction, WMC and atrophy were dichotomised as none/mild versus moderate/severe. For patients who had undergone both MRI and CT brain imaging, the MRI scans were used in the analyses. To determine the association between WMC and delirium, and atrophy and delirium, we calculated odds ratios (ORs) adjusted for age and sex (model 1), age, sex, baseline stroke severity, depression and illness severity as defined by SIRS (model 2), age, sex, baseline stroke severity, depression and illness severity, pre-admission MMSE (model 3) and age, sex, baseline stroke severity, depression and illness severity and pre-admission MoCA (model 4). For patients with multiple admissions, we used data from the first admission episode only. Similar analyses were performed after exclusion of patients with severe stroke (NIHSS > 10) and those whose baseline TIA/stroke brain imaging was done >5 years prior to hospital admission.

We also performed sensitivity analyses to calculate ORs for associations with delirium using the full range of WMC and atrophy severities (none, mild, moderate, severe). In addition, we performed linear regression analyses with the number of delirium episodes adjusted for the number of admissions. Adjustment for covariates was done as for the logistic regression analyses, but illness severity was omitted from models 2 to 4 as this varied between admissions.

### Data Availability Statement

Applications for access to study data will be considered by PMR(peter.rothwell@ndcn.ox.ac.uk).

## Results

Among TIA and stroke patients recruited since April 2002 (mean ± SD age at index event 68.9 ± 13.3, range 21–102 years, 751 female, 676 TIA) 1,565 were still alive on 12 October 2013. Over the subsequent study period, there were a total of 194 hospital admissions (*n* = 130 single admission, *n* = 23 two admissions, *n* = 2 three admissions, *n* = 3 four admissions) in 158 OXVASC participants [mean/standard deviation (SD) age at admission = 79.2/11.5 years, range 47–100 years, 71 female]. There were 100 admissions to OUHFT and 94 admissions to EMU. In total, 170 (88%) admissions were unplanned: 122 (72%) to acute general (internal) medicine; 15 other medical; 23 surgery; 10 trauma.

Mean/SD time between the index OXVASC TIA/stroke brain imaging and hospital admission was 4.7/3.6 years and between most recent OXVASC follow-up and hospital admission was 1.8/1.8 years (Table 1). Delirium occurred in 67/194 (34.5%) admissions and 59 patients had at least one admission complicated by delirium (53 had one episode, 4 had two episodes and 2 had three episodes).

**Table 1:** Among 1,565 TIA/stroke survivors, *n* = 158 participants hospitalised over current study period: clinical and demographic factors, brain imaging and vascular risk factors, in patients with and without any delirium episode

Time to admission and demographics	Total *N* = 158	Any delirium *N* = 59	No delirium *N* = 99	*P* unadj.	*P* adj.
Time from baseline brain imaging to admission, mean/SD years	4.7/3.6	5.5/3.7	4.2/3.5	0.04	0.73
Age at admission mean/SD, years	79.3/11.5	85.3/8.7	75.7/11.6	<0.0001	<0.0001
Male sex	87 (55.1)	27 (45.8)	60 (60.6)	0.07	0.18
Education < 12 years	63 (39.9)	24 (40.7)	39 (39.4)	0.10	0.75
Brain imaging factors measured at baseline					
WMC^a^	46 (29.1)	27 (45.8)	19 (19.2)	<0.0001	0.02
Cerebral atrophy^a^	92 (58.2)	41 (69.5)	51 (51.5)	0.03	0.05
Clinical/Vascular factors measured at baseline					
NIHSS, mean/SD	2.3/4.1	2.5/4.6	2.1/4.0	0.60	0.27
History of depression	31 (19.6)	17 (28.8)	14 (14.1)	0.03	0.02
Previous stroke before index TIA/stroke event	14 (8.9)	6 (10.2)	8 (8.1)	0.67	0.16
Previous TIA before index TIA/stroke event	15 (9.5)	9 (15.3)	6 (6.1)	0.06	0.26
Hypertension	99 (62.7)	43 (72.9)	56 (56.6)	0.04	0.17
Diabetes	35 (22.2)	13 (20.3)	22 (22.2)	0.98	0.51
Hyperlipidaemia	62 (39.2)	23 (39.0)	39 (39.4)	0.96	0.86
Myocardial infarction	26 (16.5)	13 (22.0)	13 (13.1)	0.14	0.38
Atrial fibrillation	47 (29.9)	21 (35.6)	26 (26.3)	0.22	0.34
Any smoking	60 (38.0)	22 (37.3)	38 (38.4)	0.39	0.24
Current smoking	11 (7.0)	3 (5.1)	8 (8.1)	0.46	0.20
Peripheral vasc. disease	26 (16.5)	13 (22.0)	13 (13.1)	0.14	0.38
Pre-admission cognitive status at most recent OXVASC follow-up					
Dementia^b^	33 (20.9)	24 (40.7)	9 (9.1)	<0.0001	<0.0001
MMSE score, mean/SD	25.0/4.5	23.1/5.1	26.0/3.8	<0.0001	0.003
MoCA score, mean/SD	22.2/5.6	23.9/4.5	18.8/6.1	<0.0001	0.003
Illness severity at hospital admission					
SIRS, mean/SD	1.24/1.07	1.48/1.13	1.11/1.01	0.02	0.003

Numbers are *N* (%) or mean/SD. adj. = adjusted for age and sex. vasc. = vascular.

^a^Moderate/severe.

^b^OXVASC study diagnosis

Patients with versus without any delirium episode were older (mean/SD age = 85.3/8.7 versus 75.7/11.6 years, *P* < 0.0001) and had more moderate/severe WMC (27, 45.8% versus 19, 19.2%, *P* < 0.0001), atrophy (41, 69.5% versus 51, 51.5%, *P* = 0.03), depression (17, 28.8% versus 14, 14.1%, *P* = 0.03) and dementia (24, 40.7% versus 9, 9.1%, *P* < 0.0001), and worse pre-admission cognitive scores (mean/SD MoCA = 18.8/6.1 versus 23.9/4.5, *P* < 0.0001 and mean/SD MMSE = 23.1/5.1 versus 26.0/3.8, *P* = 0.0001) and severe illness during admission (mean/SD SIRS = 1.48/1.13 versus 1.11/1.01, *P* = 0.003, Table 1). There were no significant differences in sex, education, index event NIHSS or vascular risk factors.

In unadjusted logistic regression analyses, WMC were significantly associated with delirium [OR = 3.41 95% confidence interval (CI) = 1.66–7.00, *P* = 0.001] as was atrophy (OR = 2.50, 1.23–5.08, *P* = 0.01, Table 2). Associations with WMC were enhanced when only those participants admitted within 5 years of baseline brain imaging were considered and remained significant even after full adjustment including for pre-admission cognition: OR = 6.04 (2.39–15.24) *P* < 0.0001 unadjusted, and OR = 4.83 (1.29–18.13), *P* = 0.02 (model 3, adjusted for MMSE) and OR = 5.15 (1.26–21.09), *P* = 0.02 (model 4, adjusted for MoCA, Table 2). Similarly, associations with atrophy were strengthened (OR = 4.64, 1.46–14.82, *P* = 0.009, unadjusted) but attenuated after full adjustment with MMSE (OR = 1.84, 0.38–8.77, *P* = 0.45) and MoCA (OR = 2.38, 0.40–14.15, *P* = 0.34). In contrast, no associations were seen for either WMC or atrophy in patients who were admitted >5 years after their baseline brain imaging (see Appendices). Sensitivity analyses using linear regression with the number of delirium episodes adjusted for the number of admissions as the dependent variable showed similar associations between WMC and delirium, and atrophy and delirium (see Appendices).

**Table 2:** Associations between WMC and delirium and cerebral atrophy and delirium, unadjusted and adjusted models for all patients and restricted to patients with baseline brain imaging within 5 years of admission, without dementia, with TIA or less severe stroke (NIHSS < 10) and aged ≥ 75 years

	Unadjusted		Model 1 (age, sex)		Model 2 (age, sex, illness severity, NIHSS, depression)		Model 3 (age, sex, illness severity, NIHSS, depression, MMSE)		Model 4 (age, sex, illness severity, NIHSS, depression, MoCA)	
All patients										
	OR 95% CI	*P*	OR 95% CI	*P*	OR 95% CI	*P*	OR 95% CI	*P*	OR 95% CI	*P*
WMC	3.41 1.66–7.0	0.001	2.67 1.21–5.85	0.02	3.00 1.29–6.78	0.01	2.94 1.10–7.88	0.03	2.25 0.74–6.85	0.15
Cerebral atrophy	2.50 1.23–5.08	0.01	2.18 1.00–4.73	0.05	2.37 1.02–5.50	0.05	2.30 0.87–6.07	0.09	2.08 0.68–6.30	0.20
Patients admitted within 5 years of baseline imaging										
WMC	6.04 2.39–15.24	<0.0001	4.27 1.57–11.57	0.004	4.37 1.54–12.38	0.006	4.83 1.29–18.13	0.02	5.15 1.26–21.09	0.02
Cerebral atrophy	4.64 1.46–14.82	0.009	3.02 0.85–10.69	0.09	2.55 0.69–9.41	0.16	1.84 0.38–8.77	0.45	2.38 0.40–14.15	0.34
Patients without dementia										
WMC	4.94 2.01–12.08	<0.0001	3.48 1.29–9.38	0.01	4.90 1.62–14.82	0.005	4.86 1.51–15.70	0.008	2.93 0.77–11.12	0.11
Cerebral atrophy	3.65 1.43–9.32	0.007	2.74 1.00–7.55	0.05	2.67 0.91–7.95	0.07	2.43 0.78–7.55	0.13	2.01 0.56–7.25	0.28
Patients with NIHSS < 10										
WMC	3.94 1.83–8.45	<0.0001	2.81 1.21–6.53	0.02	3.43 1.37–8.54	0.008	2.38 0.89–6.53	0.09	1.80 0.56–5.84	0.33
Cerebral atrophy	2.15 1.04–4.47	0.04	1.75 0.78–3.95	0.18	2.08 0.55–5.05	0.11	2.07 0.78–5.51	0.15	1.87 0.61–5.76	0.27
Patients ≥75 years										
WMC	2.97 1.31–6.74	0.009	2.46 1.05–5.89	0.04	3.29 1.25–8.64	0.02	2.37 0.81–6.94	0.12	1.84 0.56–6.07	0.32
Cerebral atrophy	1.66 0.50–5.57	0.41	1.75 0.62–5.00	0.29	1.85 0.72–4.77	0.21	1.59 0.69–3.66	0.28	1.68 0.76–3.72	0.20

When analyses were restricted to patients without dementia, associations between delirium and both WMC and atrophy were somewhat stronger than in the cohort overall both in unadjusted analyses (OR = 4.94, 2.01–12.08, *P* < 0.0001 and 3.65, 1.43–9.32, *P* = 0.007) and after adjustment for demographic factors, illness severity and depression although associations attenuated with the addition of pre-admission cognition. Associations were also qualitatively similar in those with less severe index cerebrovascular events (NIHSS < 10) and older age (Table 2).

Looking separately at the different imaging modalities, there were 85 patients with CT only, 22 with MRI only and 51 with both CT and MRI. Patients with CT only were older (mean/SD age = 81.0/12.1 years) than those with MRI (76.1/11.5 years, *P* = 0.03). For patients admitted within 5 years after baseline imaging, MRI-defined WMC were associated with delirium (OR = 9.28, 2.27–38.0, *P* = 0.002) but associations with CT-defined WMC were less strong and just failed to reach significance (OR = 3.33, 0.92–12.11, *P* = 0.07, Table 3). Similarly, atrophy on MRI was associated with delirium (OR = 8.50, 1.00–71.71, *P* = 0.05), whereas CT-defined atrophy did not reach significance (OR = 5.10, 0.95–27.4, *P* = 0.06).

**Table 3:** Associations between WMC and delirium, and cerebral atrophy and delirium, unadjusted and adjusted for patients ≤5 years since baseline brain imaging, stratified by CT versus MRI

	Unadjusted		Model 1 (age, sex)		Model 2 (age, sex, illness severity, NIHSS, depression)		Model 3 (age, sex, illness severity, NIHSS, depression, MMSE)		Model 4 (age, sex, illness severity, NIHSS, depression, MoCA)	
	OR 95% CI	*P*	OR 95% CI	*P*	OR 95% CI	*P*	OR 95% CI	*P*	OR 95% CI	
Moderate/severe WMC										
CT	3.33 0.92–12.11	0.07	2.48 0.61–10.07	0.21	3.47 0.64–18.77	0.15	1.58 0.17–14.90	0.69	1.14 0.11–11.98	0.90
MRI	9.28 2.27–37.0	0.002	6.91 1.55–30.85	0.01	8.67 1.56–48.2	0.01	21.42 1.46–313.55	0.03	12.40 0.82–187.6	0.07
Cerebral atrophy										
CT	5.10 0.95–2.74	0.06	3.60 0.59–22.1	0.17	2.59 0.36–18.36	0.34	2.16 0.14–32.10	0.58	-	-
MRI	8.50 1.00–71.71	0.05	6.58 0.71–61.41	0.10	6.83 0.52–90.50	0.15	6.40 0.24–172.30	0.27	5.44 0.24–125.2	0.29

Sensitivity analyses using the full range of WMC and atrophy severity scores (none, mild, moderate, severe) to calculate ORs for associations with delirium showed similar findings to when the dichotomised scores (none/mild versus moderate/severe) were used (see Appendices).

## Discussion

Brain imaging acquired in the course of standard care for older patients may have clinical utility beyond its immediate indication. Our study, a large prospective study of brain imaging and delirium on long-term follow-up, examined the predictive value of routinely acquired imaging data for delirium occurring years later. In our unselected TIA/stroke survivors, severity of white matter disease, and to a lesser extent cerebral atrophy, on brain imaging predicted delirium during hospitalisation occurring up to 5 years later. The predictive value of the prior imaging findings was maintained when only patients without dementia or those with minor cerebrovascular events were considered.

A recent systematic review of the imaging correlates of delirium found evidence for associations with both WMCs and atrophy as well as alterations in cerebral blood flow and metabolism [22]. Most previous data derive from selected cohorts in which brain imaging, often using research protocols, was done shortly before elective admission [23–27]. Our findings from an unselected cohort suggest that the delirium risk conferred by WMC occurs over and above its impact on pre-existing cognition. Cerebrovascular disease damages the blood brain barrier, which may facilitate entry of systemic inflammatory factors to the brain [28]. Inflammation is associated with small vessel disease [29] and deterioration in white matter integrity in fronto-temporal/limbic regions may occur following delirium suggesting that systemic inflammation may be important [30]. Predisposition to delirium might explain why the relative prevalence of vascular versus other dementia in hospitalised patients is higher than in the background population [17].

Although we found associations with global atrophy, these were less robust than associations with WMC and largely disappeared after adjustment for pre-admission cognition suggesting that global atrophy, at least as measured through expert radiologist review rather than application of specific atrophy visual rating scales, may not be an independent risk factor. Global atrophy has been inconsistently linked with delirium in previous studies [23, 24, 31], although associations with specific regional (temporal/limbic) atrophy have been reported [32, 33]. Cerebral atrophy occurs in neurodegeneration but is not pathognomic and may occur in, for example, cerebrovascular disease, head injury [34], demyelination [35] and alcohol excess [36], but disproportionate temporal lobe atrophy is generally considered characteristic of Alzheimer’s disease [37]. We were unable to examine associations between temporal lobe atrophy and delirium since routinely acquired CT and MRI brain scans are not optimised to facilitate temporal lobe/hippocampal atrophy quantification. Neither amyloid positron emission tomography imaging [32] nor autopsy studies provide supportive evidence [38] for a role for Alzheimer’s disease in delirium, and memory clinic patients with vascular cognitive impairment appear more susceptible to delirium [39], but data are limited.

Our findings of less predictive power for CT-defined moderate/severe WMC versus MRI should be interpreted with caution and require further study. Patients with CT-only were older than those with MRI and since white matter disease is less associated with cognition with increasing age [40], it might also be less strongly related to delirium risk. This might not be a major issue from a clinical perspective since identification of younger patients at high risk of delirium is likely to be more useful in individualised management than in older old patients in whom the majority will be at risk.

Our findings have potential clinical utility because prior brain imaging could aid risk stratification for delirium in emergency admissions and inform risk/benefit considerations in elective procedures. Addition of brain imaging data, using standardised radiology reporting, might strengthen existing delirium risk prediction models and with the development of reliable artificial intelligence brain imaging analysis could enable automated risk prediction in digital systems [8, 41]. The ability of brain imaging to detect loss of cerebral reserve as manifested by brain vulnerability to delirium over and above premorbid cognition further highlights its potential clinical utility particularly since pre-admission cognitive status is often unknown [6, 7, 42]). Finally, brain imaging might also provide prognostic information regarding delirium duration and severity and, importantly, future risks of death and dementia. Although delirium appears to accelerate cognitive decline [43], whether imaging can predict cognitive trajectory at an individual level remains uncertain.

Strengths of our study include the nesting within an ongoing longitudinal population-based cohort study with prospective patient evaluation for delirium in which the prior OXVASC study consent/assent facilitated inclusive ascertainment. Baseline brain imaging was done routinely on all OXVASC patients thus avoiding the selection bias inherent in using patients with brain imaging from general cohorts. The study design also allowed robust adjustment for confounders including previous cognitive function and illness severity. In addition, we included CT-based imaging so that data were available on frail older patients unable to tolerate MRI.

Limitations include the possibility of survival bias with at-risk patients dying in the interval between brain imaging and hospital admission reducing the strength of observed associations. We did not use a validated visual rating scale for atrophy or specifically consider temporal lobe atrophy and although our use of simple dichotomised measures of WMC and atrophy enhanced potential clinical utility, it may have impacted our ability to show associations. We were unable to directly compare the predictive value of MRI versus CT as the two modalities were not performed together in sufficient patients, although our previous studies showed good agreement in grading of white matter severity [10]. Finally, delirium diagnosis was made by a single clinician on the basis of the DSM-IV criteria after review of all available evidence but specific tests of attention such as that contained within the 4AT [44] were not performed. Particular challenges arise in diagnosing delirium in patients with previous cognitive impairment [45], but we reviewed information from the collateral history, in particular for evidence of change in behaviour, or conscious level where possible.

In conclusion, a high WMC burden and atrophy on standard clinical brain imaging predict long-term delirium risk. Since WMC are more prevalent in small vessel disease stroke (although not large artery or cardioembolic stroke) than in matched controls [46], further work is required to assess the generalisability of our findings to non-cerebrovascular cohorts. Finally, future studies should address the question of whether findings from routinely acquired brain imaging influence delirium prognosis including its severity and duration and longer-term cognitive outcomes.

## Supplementary Material

aa-21-0388-File003_afab200
