# Supplementary material for: Utility of white matter disease and atrophy on routinely acquired brain imaging for prediction of long-term delirium risk: population-based cohort study
Source: Age Ageing. 2021 Nov 13;51(1):afab200. doi: 10.1093/ageing/afab200 (PMC8753040; doi:10.1093/ageing/afab200)
Supplement: aa-21-0388-File003_afab200 [file aa-21-0388-file003_afab200.docx]

APPENDICES

Utility of white matter disease and atrophy on routinely acquired brain imaging for prediction of long-term delirium risk: population-based cohort study

ST Pendlebury FRCP DPhil,^1-3^ RJ Thomson BM BCh,^4^ SJV Welch RGN,^1^ W Kuker MD,^1^ PM Rothwell FMedSci^1^ for the Oxford Vascular Study

1. Wolfson Centre for Prevention of Stroke and Dementia, Wolfson Building, Nuffield Department of Clinical Neurosciences, John Radcliffe Hospital, and the University of Oxford

2. NIHR Oxford Biomedical Research Centre, Oxford University Hospitals NHS Foundation Trust.

3. Departments of General (Internal) Medicine and Geratology, Oxford University Hospitals NHS Foundation Trust.

4. Department of Cardiology, Royal Free Hospital, London.

Correspondence to:

Professor Sarah Pendlebury

Wolfson Centre for Prevention of Stroke and Dementia

Wolfson Building,

John Radcliffe Hospital

Oxford OX3 9DU

Tel: +44 1865 231603

Email: [sarah.pendlebury@ndcn.ox.ac.uk](mailto:sarah.pendlebury@ndcn.ox.ac.uk)

Appendices Contents Page

Appendix Methods 3

Appendix Tables 5-8

Appendix Table 1 5

Associations between WMC and delirium, and between atrophy and delirium for patients admitted >5 years after baseline brain imaging.

Appendix Table 2 6

Associations between severity of WMC (graded 0-3) and delirium, and severity of cerebral atrophy (graded 0-3) and delirium.

Appendix Table 3 7

Associations between severity of WMC (graded 0-3) and delirium, and severity of cerebral atrophy (graded 0-3) and delirium stratified by CT versus MRI.

Appendix Table 4 8

Linear regression (outcome=number of delirium episodes, adjusted for number of admissions), showing associations between WMC and delirium, and atrophy and delirium.

Appendix Methods

Ascertainment of delirium

The AMTS [1] is done routinely in OUHFT as it is helpful to know the level of a patient’s cognitive function whether or not they have delirium. Many patients are not delirious but may nevertheless be impaired because of dementia (which may be undiagnosed) or mild or transient cognitive impairments. In addition, documentation of a normal AMTS may be helpful in establishing the patient’s baseline (accepting that the AMST is specific but not overly sensitive for cognitive impairments [2]). The AMTS is not itself diagnostic of delirium but it does inform the application of the Confusion Assessment Method-CAM [3]. The AMTS allows identification of a cognitive deficit and its severity, but the next step is to determine whether this is caused by delirium, dementia, delirium superimposed on dementia or something else. OUHFT training around delirium highlights the need to use information from the clinical assessment process to decide whether the CAM screen is positive and then whether a clinical diagnosis of delirium can be made according to the DSM-IV (now DSM-5) criteria.

The CAM was developed as an operationalisation of the DSM criteria [3] and was designed to be used during the process of routine patient assessment. Specifically, the CAM criteria are assessed using information gained during the clerking or subsequent medical or MDT assessments as follows:

A. Acute onset/fluctuation – obtained from a detailed collateral history. This is particularly important where a patient has a per-existing cognitive impairment where the change from the previous level of function and behaviour may be key to the recognition of delirium.

B. Inattention- Does the patient have difficulty focusing attention, is the patient easily distractible? Do they have difficulty keeping track of what is being said? Did the patient struggle with 20-1 backwards doing the AMTS or in keeping their mind on the questions?

C. Disordered thinking –. Is the patient’s train of thought muddled or illogical? Is it hard to follow what they are saying?

D Altered level of consciousness - Is the patient sleepy, less interactive than usual, or agitated (eg walking around the ward, interfering with equipment, picking at clothes/drips/bedclothes). This is another key aspect of delirium that aids its identification in patients with a pre-existing cognitive impairment. Informants will be often be very skilled at picking up such changes.

In practice, criterion C is most challenging but criterion D is usually more easily assessed. To screen positive patients must have A and B and either C or D

The diagnosis of delirium may be particularly difficult in patients with pre-existing cognitive impairment. At present, there is no consensus on how best to evaluate these patients [4] but, criteria A and D from the CAM may be particularly useful and a detailed collateral history must be obtained.

References

1. Hodkinson HM. Evaluation of a mental test score for assessment of mental impairment in the elderly. Age Ageing 1972;1:233-238.

2. Emery A, Wells J, Klaus SP, Mather M, Pessoa A, Pendlebury ST. Underestimation of Cognitive Impairment in Older Inpatients by the Abbreviated Mental Test Score versus the Montreal Cognitive Assessment: Cross-Sectional Observational Study. Dement Geriatr Cogn Dis Extra. 2020 Dec 15;10(3):205-215.

3. Inouye SK, van Dyck CH, Alessi CA, Balkin S, Siegal AP, Horwitz RI. Clarifying confusion: the confusion assessment method. A new method for detection of delirium. Ann Intern Med 1990;113:941-948.

4. Morandi A, Davis D, Bellelli G, Arora RC, Caplan GA, Kamholz B, Kolanowski A, Fick DM, Kreisel S, MacLullich A, Meagher D, Neufeld K, Pandharipande PP, Richardson S, Slooter AJ, Taylor JP, Thomas C, Tieges Z, Teodorczuk A, Voyer P, Rudolph JL. The Diagnosis of Delirium Superimposed on Dementia: An Emerging Challenge. J Am Med Dir Assoc. 2017;18:12-18.

Appendix Table 1. Associations between WMC and delirium, and between atrophy and delirium for patients admitted >5 years after baseline brain imaging.

|  | Unadjusted | | Model 1 (age, sex) | | Model 2 (age, sex, illness severity, NIHSS, depression) | | Model 3 (age, sex, illness severity, NIHSS, depression, MMSE) | | Model 4 (age, sex, illness severity, NIHSS, depression, MoCA) | |
| --- | --- | --- | --- | --- | --- | --- | --- | --- | --- | --- |
|  | OR  95% CI | p | OR  95% CI | p | OR  95% CI | p | OR  95% CI | p | OR  95% CI | p |
| Moderate/severe WMC | 1.78  0.52-6.12 | 0.36 | 1.72  0.42-7.03 | 0.45 | 1.63  0.38-6.97 | 0.51 | 2.04  0.45-9.19 | 0.36 | 0.41  0.05-3.63 | 0.42 |
| Cerebral atrophy | 1.64  0.58-4.65 | 0.35 | 1.62  0.52-5.09 | 0.41 | 1.58  0.42-5.86 | 0.50 | 2.38  0.48-11.79 | 0.29 | 1.03  0.13-8.38 | 0.98 |

WMC=White matter changes

Appendix Table 2. Associations between severity of WMC (graded 0-3)*, and delirium and severity of cerebral atrophy (graded 0-3)* and delirium, unadjusted and adjusted models for all patients and restricted to patients with baseline brain imaging within 5 years of admission, without dementia, with TIA or less severe stroke (NIHSS<10) and aged>75 years. Odds ratios are per point increase in severity grading.

|  | Unadjusted | | Model 1 (age, sex) | | Model 2 (age, sex, illness severity, NIHSS, depression) | | Model 3 (age, sex, illness severity, NIHSS, depression, MMSE) | | Model 4 (age, sex, illness severity, NIHSS, depression, MoCA) | |
| --- | --- | --- | --- | --- | --- | --- | --- | --- | --- | --- |
| All patients | | | | | | | | | | |
|  | OR  95% CI | p | OR  95% CI | p | OR  95% CI | p | OR  95% CI | p | OR  95% CI | p |
| WMC | 1.90  1.35-2.68 | <0.001 | 1.57  1.09-2.28 | 0.02 | 1.60  1.09-2.35 | 0.02 | 1.58  0.97-1.15 | 0.07 | 1.55  0.89-2.71 | 0.12 |
| Cerebral atrophy | 1.49  1.07-2.09 | 0.02 | 1.35  0.93-1.95 | 0.12 | 1.30  0.89-1.92 | 0.18 | 1.33  0.86-2.08 | 0.20 | 1.27  0.77-2.11 | 0.35 |
| Patients admitted within 5 years of baseline imaging | | | | | | | | | | |
| WMC | 2.45  1.53-3.93 | <0.001 | 2.06  1.26-3.38 | 0.004 | 2.03  1.21-3.40 | 0.007 | 2.56  1.26-5.20 | 0.009 | 2.58  1.21-5.52 | 0.02 |
| Cerebral atrophy | 1.63  1.03-2.60 | 0.04 | 1.25  0.74-2.11 | 0.40 | 1.15  0.65-1.92 | 0.69 | 1.15  0.60-2.22 | 0.68 | 1.39  0.67-2.91 | 0.38 |
| Patients without dementia | | | | | | | | | | |
| WMC | 2.09  1.35-3.23 | 0.001 | 1.60  0.98-2.61 | 0.06 | 1.83  1.07-1.31 | 0.03 | 1.78  1.00-3.17 | 0.05 | 1.69  0.87-3.30 | 0.13 |
| Cerebral atrophy | 1.84  1.19-2.84 | 0.006 | 1.53  0.95-2.48 | 0.08 | 1.55  0.92-2.59 | 0.10 | 1.50  0.87-2.58 | 0.14 | 1.31  0.72-2.41 | 0.38 |
| Patients with NIHSS<10 | | | | | | | | | | |
| WMC | 2.04  1.41-2.96 | <0.001 | 1.62  1.08-2.42 | 0.02 | 1.75  1.13-2.70 | 0.01 | 1.42  0.86-2.36 | 0.18 | 1.35  0.75-2.42 | 0.31 |
| Cerebral atrophy | 1.45  1.01-2.07 | 0.04 | 1.26  0.84-1.87 | 0.27 | 1.30  0.85-1.99 | 0.22 | 1.30  0.83-2.05 | 0.25 | 1.20  0.72-2.01 | 0.48 |
| Patients >75 years | | | | | | | | | | |
| WMC | 1.72  1.16-2.56 | 0.007 | 1.52  1.01-2.30 | 0.05 | 1.71  1.07-2.72 | 0.03 | 1.45  0.83-2.56 | 0.20 | 1.40  0.75-2.61 | 0.29 |
| Cerebral atrophy | 1.28  0.88-1.88 | 0.20 | 1.24  0.83-1.85 | 0.30 | 1.25  0.81-1.94 | 0.32 | 1.25  0.78-2.02 | 0.35 | 21.19  0.69-2.05 | 0.53 |

*0=none, 1=mild, 2=moderate, 3=severe

Appendix Table 3. Associations between delirium, and severity of WMC (graded 0-3)*, and delirium and severity of cerebral atrophy (graded 0-3)*, unadjusted and adjusted for patients <5-years since baseline brain imaging, stratified by CT versus MRI. Odds ratios are per point increase in severity grading.

|  | Unadjusted | | Model 1 | | Model 2 (age, sex, illness severity, NIHSS, depression) | | Model 3 (age, sex, illness severity, NIHSS, depression, MMSE) | | Model 4 (age, sex, illness severity, NIHSS, depression, MoCA) | |
| --- | --- | --- | --- | --- | --- | --- | --- | --- | --- | --- |
|  | OR  95% CI | p | OR  95% CI | p | OR  95% CI | p | OR  95% CI | p | OR  95% CI | |
| WMC | | | | | | | | | | |
| CT | 2.56  1.27-5.18 | 0.009 | 2.20  1.06-4.56 | 0.04 | 2.60  1.04-6.52 | 0.04 | 2.87  0.79-10.50 | 0.11 | 2.59  0.69-9.77 | 0.16 |
| MRI | 2.47  1.27-4.79 | 0.008 | 2.08  1.02-4.34 | 0.04 | 2.23  1.00-5.00 | 0.05 | 4.90  1.11-21.68 | 0.04 | 3.41  0.85-13.72 | 0.09 |
| Cerebral atrophy | | | | | | | | | | |
| CT | 1.22  0.64-2.34 | 0.55 | 0.90  0.42-1.93 | 0.79 | 0.53  0.20-1.39 | 0.20 | 0.49  0.15-1.69 | 0.26 | 0.75  0.19-3.01 | 0.68 |
| MRI | 2.30  1.07-4.92 | 0.03 | 1.88  0.84-4.22 | 0.13 | 1.97  0.78-4.95 | 0..15 | 0.92  0.75-1.11 | 0.38 | 1.84  0.52-6.53 | 0.35 |

*0=none, 1=mild, 2=moderate, 3=severe

Appendix Table 4. Linear regression (outcome=number of delirium episodes, adjusted for number of admissions), showing associations between WMC and delirium, and atrophy and delirium, for all patients, those with NIHSS<10, and patients admitted <5 years and >5 years after baseline brain imaging.

|  | Unadjusted | | Model 1 (age, sex) | | Model 2 (age, sex, NIHSS, depression) | | Model 3 (age, sex, NIHSS, depression, MMSE) | | Model 4 (age, sex, NIHSS, depression, MoCA) | |
| --- | --- | --- | --- | --- | --- | --- | --- | --- | --- | --- |
| All patients | | | | | | | | | | |
|  | Beta |  | Beta | p | Beta | p | Beta | p | Beta | p |
| Moderate/severe WMC | 0.268 | <0.0001 | 0.209 | 0.003 | 0.271 | <0.0001 | 0.218 | 0.007 | 0.156 | 0.07 |
| Cerebral atrophy | 0.151 | 0.04 | 0.102 | 0.15 | 0.081 | 0.29 | 0.069 | 0.38 | 0.046 | 0.57 |
|  | | | | | | | | | | |
| Patients with NIHSS<10 only | | | | | | | | | | |
|  | Beta |  | Beta | p | Beta | p | Beta |  | Beta | Beta |
| Moderate/severe WMC | 0.338 | <0.0001 | 0.257 | 0.001 | 0.271 | <0.0001 | 0.218 | 0.007 | 0.156 | 0.07 |
| Cerebral atrophy | 0.164 | 0.04 | 0.099 | 0.19 | 0.081 | 0.29 | 0.069 | 0.38 | 0.046 | 0.57 |
|  | | | | | | | | | | |
| All patients admitted within 5 years of baseline imaging | | | | | | | | | | |
| Moderate/severe WMC | 0.401 | <0.0001 | 0.308 | <0.0001 | 0.271 | <0.0001 | 0.218 | 0.007 | 0.156 | 0.07 |
| Cerebral atrophy | 0.319 | <0.0001 | 0.218 | 0.009 | 0.081 | 0.29 | 0.069 | 0.38 | 0.046 | 0.57 |
|  | | | | | | | | | | |
| All patients admitted >5 years after baseline imaging | | | | | | | | | | |
| Moderate/severe WMC | 0.112 | 0.24 | 0.096 | 0.30 | 0.080 | 0.37 | 0.116 | 0.23 | 0.021 | 0.82 |
| Cerebral atrophy | -0.115 | 0.22 | -0.114 | 0.22 | -0.171 | 0.07 | -0.099 | 0.38 | -0.30 | 0.75 |
